# Antibody-Mediated Therapy against HIV/AIDS: Where Are We Standing Now?

**DOI:** 10.1155/2018/8724549

**Published:** 2018-06-03

**Authors:** Noel Jacques Awi, Sin-Yeang Teow

**Affiliations:** Department of Medical Sciences (DMS), School of Healthcare and Medical Sciences (SHMS), Sunway University, Jalan Universiti, Bandar Sunway, 47500 Subang Jaya, Selangor Darul Ehsan, Malaysia

## Abstract

Acquired immunodeficiency syndrome (AIDS) cases are on the rise globally. To date, there is still no effective measure to eradicate the causative agent, human immunodeficiency virus (HIV). Highly active antiretroviral therapy (HAART) is being used in HIV/AIDS management, but it results in long-term medication and has major drawbacks such as multiple side effects, high cost, and increasing the generation rate of escape mutants. In addition, HAART does not control HIV-related complications, and hence more medications and further management are required. With this, other alternatives are urgently needed. In the past, small-molecule inhibitors have shown potent antiviral effects, and some of them are now being evaluated in clinical trials. The challenges in developing these small molecules for clinical use include the off-target effect, poor stability, and low bioavailability. On the other hand, antibody-mediated therapy has emerged as an important therapeutic modality for anti-HIV therapeutics development. Many antiviral antibodies, namely, broad neutralizing antibodies (bnAbs) against multiple strains of HIV, have shown promising effects* in vitro* and in animal studies; further studies are ongoing in clinical trials to evaluate their uses in clinical applications. This short review aims to discuss the current development of therapeutic antibodies against HIV and the challenges in adopting them for clinical use.

## 1. Current HIV Treatments

The discovery of HAART in 1996 has successfully reduced the global mortality rate due to HIV/AIDS [1]. This therapeutic strategy targets multiple viral proteins that are essential for viral replication and dissemination (e.g., envelope protein, reverse transcriptase, integrase, and protease), instead of targeting a single target. This combination therapy could potently suppress the viral load to minimal level. However, the treatment cost is extremely high and this therapy often results in numerous side effects that significantly affect the patients' life quality [2]. Depending on the conditions, the mild side effects could be weight loss, diarrhoea, anaemia, constipation, dizziness, insomnia, headache, fatigue, rashes, abdominal pain, emotions, nausea, and vomiting, while the potentially fatal complications are liver failure, lipodystrophy, and neurological and cardiovascular diseases [2]. In addition to physical health, other problems in mental and social health have also been reported [2]. It has also been shown that the use of HAART leads to long-term medication of HIV/AIDS patients, and it could introduce multiple drug-resistant escape mutants due to the high mutation rate and recombination frequency of the virus [3]. A large number of drug-resistant HIV variants have been previously reported, and they are known to be resistant to various drug groups such as nucleoside reverse transcriptase inhibitors (NRTIs), nonnucleoside reverse transcriptase inhibitors (NNRTIs), protease inhibitors, integrase inhibitors, and entry inhibitors [3]. Hence, other more effective and less toxic alternatives are urgently needed to treat HIV/AIDS. A proper use of anti-HIV drug is also important in order to control the overwhelming number of escape mutants.

In the past, small-molecule inhibitors have demonstrated promising antiviral effects against HIV [4–7]. These inhibitors target various viral proteins such as integrase [8], nucleocapsid [9], capsid [10], and envelope [11] as well as cellular proteins of target cells such as CD4 [12], kinases [13], and cellular cofactor [14]. T20 peptide (Enfuvirtide) is the first HIV entry inhibitor approved by United States Food and Drug Administration (U. S. FDA) in 2003 [15] followed by the approval of the first chemokine receptor 5 (CCR5) small-molecule antagonist, Maraviroc (Selzentry) in 2007 [16]. However, these drugs have their limitations in the clinical applications [6, 17]. Meanwhile, other small-molecule inhibitors are being evaluated in clinical trials. For instances, small-molecule NNRTIs, cabotegravir, and rilpivirine were evaluated in randomised phase 2b trials and the result of the trial has been recently published [18]. Following Maraviroc, small-molecule CCR5 antagonists, aplaviroc, and vicriviroc are currently being evaluated in phase 2b clinical trials for HIV therapy [19]. A small-molecule ABX464 that inhibits HIV replication is also expected to enter phase 2b trials in 2018 [20]. Despite the advancement of biological and chemical engineering technology, it remains a huge challenge to deliver a safe, stable, and functional molecule into the host system to completely eradicate the residing virus [6].

Antibody-based therapies possess several advantages over the chemical-based treatment in various aspects such as specificity and safety [21]. A number of therapeutic antibodies have been approved by FDA for cancer therapies [22]. In the past three years, therapeutic antibodies targeting programmed death protein 1 (PD-1) and programmed death ligand 1 (PDL1) to boost human immune responses against cancer cells [23] have been approved by FDA to treat various cancers including melanoma [24], non-small cell lung cancer (NSCLC) [25], and head and neck squamous cell carcinoma (HNSCC) [26]. For viral infection prevention and treatment, several antibodies targeting multiple types of viruses such as human cytomegalovirus (HCMV), influenza, respiratory syncytial virus (RSV), Ebola, rabies, and HIV are being evaluated in clinical trials [21]. The development of therapeutic antibodies, in particular, the broad neutralizing antibodies (bnAbs) against various HIV strains, will be emphasized and discussed in later sections.

## 2. Development of HIV-1-Neutralizing Antibodies against HIV/AIDS

Broad neutralizing antibodies (bnAbs) are neutralizing antibodies that neutralize multiple HIV-1 viral strains by targeting conserved epitopes of virus [60]. BnAbs have shown promising effects against various HIV strains and have significantly contributed to HIV vaccine development [60]. The earliest bnAb, b12 were discovered in 1991 [61], and increasing bnAbs have been seen in recent years [60]. All of the bnAbs information is freely available on a website known as broadly neutralizing antibodies electronic resource (bNAber) which aims to support the researchers to generate a potent HIV vaccine [62].  Table 1 shows a list of bnAbs targeting multiple HIV-1 epitopes in the past 10 years. These bnAbs were raised from blood of HIV-1 infected patients and this information is tabulated in Table 1. Generally, anti-HIV bnAbs can be categorized into two groups: first and second [63]. Both of these groups differ from the method used for generation of bnAbs and their functionalities. Examples of “first-generation” antibodies are b12, 2G12, 4E10, and 2F5. These bnAbs were developed from Epstein-Barr virus- (EBV-) immortalized B cells and generated using phage-display methods [63]. Although these bnAbs resulted in 50%–88% of neutralization breadth, they are not ideal for antibody-based vaccine mainly due to the relatively low neutralization efficacy and limitation of method such as antibody specificity selection and lack of high throughput screening option [64]. On the other hand, “second-generation” bnAbs possess improved neutralization potency and offer high flexibility in technology manipulation. The advancement of technology allows the selection of chronically HIV-1-infected “Elite neutralizers,” new screening and selection method, and new antibody isolation and analysis method [64]. Examples of “second-generation” bnAbs are PG9, PG16, CH01, PGT145, PGT121, PGDM1400, 10-1074, 10E8, VRC01, 3BNC117, and CH103.

To date, none of the FDA-approved therapeutic antibodies are used for HIV treatment. The development of therapeutic antibodies against HIV is ongoing and many of them have successfully progressed to human clinical trials [46, 65].  Table 2 shows the anti-HIV antibodies that are being evaluated in different phases of trials. HIV-1 envelope protein is a popular therapeutic target for various antiviral pharmaceuticals and vaccine design [66]. Scheid and group demonstrated the result of phase 2a trial using HIV Env-specific antibody, 3BNC117 on 13 HIV-infected patients. They showed that the broad neutralizing antibody (bnAb) 3BNC117 exerted strong selective pressure on HIV-1 emerging from latent reservoirs [43]. Similarly, another monoclonal antibody, 10-1074 targeting V3 glycan site of HIV-1 Env protein, showed potent effect and high tolerant limit in phase I trial [44]. Bar and colleagues showed another bnAb, VRC01 to effectively suppress the plasma viremia below detectable concentration in phase I trial [45]. The same study also highlighted the emergence of VRC01-resistant HIV after the exposure as the main challenge [45]. Other monoclonal antibodies targeting HIV envelope glycoproteins that are currently under phase I trials are PGDM1400, PGT121, N6, and 10E8v4 (Table 2) [46]. Recently, Xu and group generated highly potent trispecific antibodies by combining the specificity of PGDM1400, VRC01, and 10E8v4 [47]. These engineered antibodies interact with three different sites of envelope protein: membrane-proximal external region (MPER)-, CD4-, and V1V2-binding sites. These antibodies conferred a complete immunity by displaying high breadth and potency against simian-human immunodeficiency viruses (SHIVs) in nonhuman primates compared to each parental antibody. This antibody developed by Sanofi is expected to enter phase I trial in late 2018 [47].

Antibodies that are targeting cellular proteins essential for HIV replication such as CD4 and coreceptors have also been shown to efficaciously eradicate the HIV. Some of these antibodies are undergoing clinical trials. TMB-355 (previously known as TMB-301), also known as ibalizumab, targets CD4 receptor and prevents the viruses from binding to them. A phase 3 trial demonstrated that ibalizumab is a safe and well-tolerated drug and can be used as a monotherapy especially for multidrug-resistant HIV patients with limited treatment options [51]. Researchers are currently developing a new formulation of ibalizumab for the intramuscular injection use [51]. Various antibodies targeting chemokine coreceptor 5 (CCR5), such as HGS004, PRO140, and Cenicriviroc (Table 2), have shown promising antiviral effects and are now being evaluated in clinical trials. An anti-PDL1 antibody, BMS-936559 has been evaluated in a phase I trial on HIV patients, and the results showed that the immunologic checkpoint inhibitor could enhance the HIV-specific immunity in a subset of participants in the trial [52]. Apart from the abovementioned antibodies, other anti-HIV therapeutic antibodies are being evaluated and these antibodies have been discussed in several reviews [59, 67–69]. These investigational therapeutic antibodies are being evaluated preclinically before entering the human trials.

Compared with other chemical-based or small-molecule treatment, antibody-based treatment offers a more comprehensive targeting and neutralization, and hence a more potent anti-HIV actions. One of the key components of bnAbs is the IgG Fc region. Using humanized mouse models, Halper-Stromberg et al. demonstrated that the HIV suppression by the passively transferred antibodies was dependent on interaction of the IgG Fc with Fc receptors of immune cells [70]. Other studies have also highlighted the Fc receptor-mediated effector functions of bnAbs in HIV inhibition through antibody-dependent cell-mediated cytotoxicity (ADCC) against both free virus and virus-infected cells [71, 72]. Fc domains of antibodies also interacted with natural killer cells and phagocytes for viral particle clearance and killing of virus-infected cells [73]. It is clear that while reactivity and affinity of bnAbs play pivotal roles in determining the antibody neutralization capacity, the Fc-mediated effector mechanisms also play part in the antiviral activities.

## 3. Challenges in Antibody-Based Therapies

As discussed above, antiviral antibodies serve as a highly potential therapeutic modality against HIV. However, there are multiple challenges in the development of these antibodies. These challenges have intensively slowed down the FDA approval of the anti-HIV antibodies into the market. This section discusses the challenges in developing the antibodies into clinical use (summarized in Table 3).

The main challenge of antibody therapy against HIV is the method used for humanized antibody production. Phage-displayed library is the most popular platform used to generate therapeutic human antibodies in the past decade and many of these phage-display-generated antibodies have been approved by FDA or European Medicines Agency (EMA) [53, 74]. This technique enables the construction of antibodies in Fab or scFv fragments and display on filamentous phage. The antibodies are then isolated and enriched by panning against antigen of interest. Due to the relatively lower throughput, phage-display technique has been switched to cell sorting and RT-PCR for antibody isolation. Use of these methods has generated many more bnAbs known as “second-generation” bnAbs. Various sources have been used for the therapeutic antibody production against viruses, including total B cells [75], single memory B cells [21], and single plasma B cells [21]. Several reports have also shown the efficacy of isolating the antibodies from native phage libraries which possess potent neutralization activities against viruses [76]. The downside of this technique is that it cannot be used to identify new neutralizing epitopes of virus [21]. This method has also been described as a time-consuming and laborious approach [77]. Other alternatives to generate the therapeutic antibodies include proteomics-directed cloning of antibodies [78] and deep sequencing of paired antibodies encoding genes [79].

The development of antibody generation technique is also highly associated with the production cost. The advancement of technologies allows the production of recombinant antibodies with improved therapeutic effects as well as other refined properties including tissue delivery and stability. For instance, antibody in single-domain antibodies (sdAbs) format allows an efficient target binding and rapid tissue penetration compared to the conventional full-length antibodies; this may enhance the antiviral activities [80]. However, this strategy needs to be reconsidered as cumulative evidences have shown the importance of Fc region of Abs in antiviral action; antibodies without Fc region would significantly reduce the overall neutralizing activities [70–72]. To improve the stability and biodistribution of therapeutic full-length antibodies and the effector functions, Fc region engineering plays an important role in improving the antibody clinical efficacy [81, 82]. Antibody-based therapies are generally well-tolerated and safe; however, this can be further improved by antibody engineering as previously described [83]. While antibody engineering is speculated to benefit the future of HIV/AIDS therapy, these modifications will increase the production cost compared to the standardized manufacturing protocol as well as the synthesis of antiviral small molecules. The production cost is the key factor for anti-HIV antibody therapy due to the smaller market in viral diseases compared to cancer diseases [21]. There is also a rising concern following the increasing number of FDA-approved small-molecule inhibitors for HIV/AIDS therapy [10, 13, 18, 20]. Although the antibody- and molecule-based therapeutics have different antiviral mechanisms, the comparison and competition between the antiviral antibodies and the substantial number of small-molecule inhibitors in the market is unavoidable.

Another challenge of developing therapeutic antibodies is the biological variation from host to host. Certain population of HIV-infected patients may not respond to a particular antibody or is suitable even though it has been proven efficacious and safe in a group of patients who have differences in terms of individuals' immunology, vaccination, diet, lifestyle, and geographical factors [21]. The treatment failure is also highly related to the biology of target virus including the mechanism of infection, mutation rate, and neutralization escape [84]. Other key challenges are the delivery and stability of antibodies for HIV therapies (Table 2). As tabulated in Table 1, all of the drug targets are viral and cellular proteins or receptors. Recent reports have highlighted the potential of targeting intracellular proteins using therapeutic antibodies for the development of HIV/AIDS therapies [85, 86]. However, conventional antibodies do not penetrate into the cells and target intracellular proteins [87]. To solve this problem, conventional antibodies can be conjugated with cell penetrating peptides (CPPs) derived from various sources to enter the cell cytoplasm [88]. Antibodies in smaller format such as Fabs, scFvs, and sdAbs (with Fc removal) have also been constructed for this purpose [57, 89, 90]. These antibodies have targeted various intracellular proteins including capsid [91], integrase [92], and other accessory proteins such as Nef [93], Vif [94], Tat [95], and Rev [96]. The clinical application of these intracellular-targeting antibodies remains to be seen.

Compared to other therapeutic modalities such as HAART and other small-molecule inhibitors, antiviral bnAbs are active against multiple types of HIV strains at low doses with acceptable toxicities [97]. For example, 3BNC117 could prevent mucosal transmission in high-dose challenge in humanized mouse models at low doses [98]. On the other hand, combination of NIH45-46m2, 10-1074, and 10E8 resulted in 100% neutralization of HIV-1 variants at a low concentration (IC_50_ of ≤0.37 *μ*g/mL) compared to the individual bnAb alone [99]. Although the toxicity of anti-HIV bnAbs therapy is less reported, it has been reported in other adjuvant therapies that may be used to complement the antibody therapy such as IL-2 and IFN-*γ* cytokine therapy [59].

Last but not least, the rapid viral resistance developed from the bnAbs treatment is striking and has drawn considerable attention. This is mainly due to the high mutation rate of HIV-1. The resistance has been reported in various bnAbs targeting multiple sites including V1/V2, V3, and CD4 binding site. Bouvin-Phey et al. reported that HIV-1 clade B has increased resistance towards the bnAbs targeting gp120 as shown by VRC01, NIH45-46, PG9, PG16, PGT121, and PGT128 [99]. Interestingly, the same study has also shown that no increase of viral resistance was seen in bnAbs targeting MPER of gp41 such as 2F5 and 4E10 [99]. This could be due to the lack of selective pressure and/or a weak tolerance to mutations in this particular region [99]. Similarly, the viral resistance of bnAbs has also been predicted using several computational and mathematical models [100, 101]. To circumvent this issue, multispecific bnAbs and combination of bnAbs have been developed to concurrently target various epitopes of a protein to reduce the chances of viral resistance. This includes the recent development of a trispecific bnAbs targeting two CD4-binding sites and MPER [102], and a combination of two to four bnAbs targeting CD4-binding site, V1V2, V3, and MPER [103].

## 4. Future Perspectives and Conclusions

The abovementioned challenges must be looked into before the therapeutic antibodies are deemed suitable for clinical use. Development of technology and bioengineering such as advancement of antibody generation and isolation methods, improvement of target delivery, and specificity play important roles in improving the antiviral activities of therapeutic antibodies. Based on current literature, many studies demonstrated that antibody-mediated antiviral treatments have yet achieved complete inhibition against HIV-1. This may suggest that treatment using therapeutic antibodies may not be suitable to be used as a “standalone” therapy although extensive efforts are ongoing. This therapeutic modality may pose a higher value when used in combination with current anti-HIV therapies such as HAART or other antiviral small-molecule inhibitors. For example, it has been shown that the bnAb 3BNC117 when used in combination with HAART could enhance the host immune response against HIV-1 and leads to significant delay of viral rebound after treatment cessation [63, 104]. Although the combination treatment of small-molecule inhibitors and antiviral antibodies has not been studied, it is speculated that it will drastically improve the neutralizing potency of the HIV-1. This speculation, however, requires further investigations for validation. In addition to maximizing the neutralization efficacy, combination treatment targeting multiple sites of protein is also anticipated to significantly avert the selection of escape variants [97].

The discovery of bnAbs has also posed a significant impact on the development of anti-HIV vaccine. While bnAbs are able to tackle the extremely high diversity of HIV-1, it is understood that having a safe vaccine eliciting bnAbs could be an effective measure to control HIV spreading. However, there are no vaccination strategies by far to elicit the effective bnAbs responses [105]. Several attempts have been made to develop the vaccination strategy mainly using animal models. In both macaque and rabbit models, sequential vaccination with gp120 and gp140 proteins has shown improved potency of neutralization, respectively [106, 107]. Similarly, sequential vaccination with three gp120 variants also demonstrated potent neutralization [108]. To date, it remains unknown to elicit the bnAbs from the HIV patients through vaccination. The main challenges are the HIV-1 diversity and later on rapid viral resistance. Sequential vaccination with various immunogens with conserved regions may promote the B cell maturation and antibody responses.

Therapeutic antibodies are known as “magic bullets” due to the fact that they can treat various diseases and infections. A number of FDA-approved therapeutic antibodies in the current market highlight the potential of adopting antibodies for disease control. Broad neutralizing antibodies have demonstrated potent antiviral action against various strains of HIV-1 and a large number of them are currently being evaluated in the clinical trials. Continuous efforts are in progress to elucidate their potential use for anti-HIV treatment in clinics. Overcoming the abovementioned challenges will undoubtedly improve the application of therapeutic antibody to HIV/AIDS management in the future.

## Figures and Tables

**Table 1:** Anti-HIV broad neutralizing antibodies (bnAbs) in the past 10 years.

Viral epitope	Name	Year	Sample source	References
V1V2 glycan site	PG9, PG16	2009	Clade A-infected African	[27]
	CH01	2011	Chronically infected donor (CH0219)	[28]
	PGT145	2011	Chronically infected donor 84	[29]
	PGDM1400	2014	Chronically infected donor 84	[30]

V3 glycan site	PGT121, PGT125, PGT135	2011	HIV-1 infected donor (at least 3 years)	[29]
	10-1074	2011	Clade A-infected African donor (patient 10)	[31]

gp41 MPER	2F5	2009	PBMCs from patient SC44	[32]
	4E10	2009	PBMCs from patient SC44	[32]
	HK20	2010	Patients plasma that showed >80% neutralization	[33]
	M66.6	2011	PBMCs from patient SC44	[32]
	CAP206-CH12	2011	Subtype C HIV-1-infected South African donor CAP206	[34]
	10E8	2012	HIV-1-infected patient N152	[35]
	m43	2012	Phage-display	[36]

CD4 binding site	VRC01	2010	PBMCs from donor 45	[37]
	HJ16	2010	Patients plasma that showed >80% neutralization	[33]
	HGN194	2010	Patients plasma that showed >80% neutralization	[33]
	3BNC117	2011	RU01 B-cell clones from patient donor	[38]
	3BNC55	2011	RU01 B-cell clones from patient donor	[38]
	VRC-PG04	2011	Clade A1/D infected donor	[39]
	VRC-CH31	2011	Chronically infected donor (CH0219)	[28]
	NIH45	2011	RU12 B-cell clones from patient donor	[38]
	8ANC195	2011	RU12 B-cell clones from patient donor	[38]
	8ANC131	2011	HIV-1 infected donor RU8	[38]
	12A12	2011	RU16 B-cell clones from patient donor	[38]
	1B2530	2011	HIV-1 infected donor RU1	[38]
	3BC176	2012	HIV-1 infected patient 3B	[40]
	3BC315	2012	HIV-1 infected patient 3B	[40]
	CH103	2013	HIV-1 infected donor CH505	[41]
	N6	2016	HIV-1-infected patient Z258	[42]

**Table 2:** Therapeutic antibodies that are being evaluated in the clinical trials.

Source	Target	Name	Clinical trial	References
Viral	Env CD4 binding site	3BNC117	Phase 2a	[43]
	Env V3 glycan site	10-1074	Phase I	[44]
	Env CD4 binding site	VRC01	Phase I	[45]
	V1V2	PGDM1400	Phase I	[42]
	Env V3 glycan site	PGT121	Phase I	[46]
	Env MPER	10E8v4	Phase I	[47]
	Env CD4 binding site	N6	Phase II	[42]

Cellular	CCR5	HGS004	Phase I	[48]
	CCR5	PRO140	Phase III	[49]
	CCR5/CCR2	Cenicriviroc	Phase III	[50]
	CD4	TMB-355	Phase III	[51]
	PDL1	BMS-936559	Phase I	[52]

**Table 3:** Challenges of FDA-approved anti-HIV therapeutic antibodies development.

Challenge	Notes	References
Method	Phage-display library is the most commonly used technique for therapeutic antibody generation.	[53]
	The technique allows both genetic and chemical modification of antibody fragments.	[54]

Cost	The production cost of antibody is higher than the synthesis of small-molecule antivirals. The generation of recombinant antibodies results in higher manufacturing cost.	[21]

Host immunology	Individuals' immune responses towards therapeutic antibodies may be different	[55]

Epidemiology	Geographical distribution affects the resistance mechanism and neutralization escape of viral strains	[56]

Delivery	Conjugation with cell-penetrating peptides improves the cellular delivery of antibodies. Smaller antibody formats such as Fabs, sdAbs, and scFvs also facilitate tissue delivery	[57]

Stability	Fc region engineering improves the half-life, stability, and the effector function of the antibody	[58]

Resistant mutants	Development of antibody-resistant HIV mutants has been reported in VRC01-treated patients, combination therapy will minimize the occurrence of virus mutation	[45]

Toxicity	IL-2 and IFN-*γ* therapy that may be used as adjuvant therapy for bnAbs therapy to stimulate T-cell killing and HIV-1 transcription resulted in high level of cytotoxicity	[59]
